# Surface enhancement of THz wave by coupling a subwavelength LiNbO_3_ slab waveguide with a composite antenna structure

**DOI:** 10.1038/s41598-017-17712-4

**Published:** 2017-12-14

**Authors:** Qi Zhang, Jiwei Qi, Qiang Wu, Yao Lu, Wenjuan Zhao, Ride Wang, Chongpei Pan, Shibiao Wang, Jingjun Xu

**Affiliations:** 1 Key Laboratory of Weak-Light Nonlinear Photonics, Ministry of Education, TEDA Institute of Applied Physics and School of physics, Nankai University, Tianjin, 300457 China; 2 Collaborative Innovation Center of Extreme Optics, Shanxi University, Taiyuan, Shanxi 030006 China

## Abstract

Highly intense terahertz electromagnetic field and efficiently surface localized terahertz field in subwavelength volumes are of vital importance for terahertz photonics integration, also will greatly accelerate the development for integrated applications in biochemical sensing, imaging, terahertz spectroscopy, enhancement of nonlinear effects and even quantum research. In this paper, we achieved large terahertz field enhancement and surface field localization through depositing a pair of Au composite antennas on a LiNbO_3_ subwavelength slab waveguide, which can serve as an excellent on-chip platform for terahertz research and application. The antennas consist of two opposing tip-to-tip triangles separated by a gap, and each triangle combines with a strip antenna. Time-resolved imaging and finite-difference time-domain method were used to resolve the characteristics of the designed antennas experimentally and simulatively. Through these methods, we demonstrated outstanding abilities of the platform: leading to a large electric field enhancement, concentrating almost full terahertz energy on the waveguide’s surface when they are resonant with the terahertz waves and tunable resonant frequency. These abilities make the subwavelength waveguide coupling with the composite antennas be able to sever as a good integrated device to identify terahertz-sensitive small objects, or an excellent platform to terahertz spectroscopy and quantum research.

## Introduction

Chip-scale photonic integrated circuits (PICs) are extremely necessary for applications such as ultra-fast telecommunications and integrated biochemical sensors^1^. Nowadays, although the PICs technology is developed at near-infrared and mid-infrared frequencies^2^, there remains an urgent demand to extend the research towards terahertz (THz) regime. THz wave is a powerful technique for sensing and spectroscopy in both research and application areas^3–5^, due to its characteristics of low photon energies (4 meV for 1 THz)^6^, the favorable resolution and information carrying capacity, as well as its fine transparency in most dielectrics. What’s more, large quantities of chemicals and molecules, such as DNA, vitamins, sugars, drugs and medicines, have characteristic absorption frequencies in terahertz regime^7,8^, which makes THz important for material identification and sensing application^9^. To achieve a universal THz integrated platform, a great deal of the interest in THz radiation increase rapidly, and various THz sources and detectors have been developed in recent years^10,11^. As an available technology enabling efficient control and manipulation of THz waves generation, THz-frequency phonon polariton (PP), who is generated in ferroelectric crystals such as LiNbO_3_ (LN) via impulsive stimulated Raman scattering (ISRS) using femtosecond laser pulses^12,13^, consequently has attracted considerable attention. Moreover, a LN slab, when its thickness becomes comparable to or less than the THz wavelength, can serve as a subwavelength planar waveguide and provide a platform for THz processing, since the generation, propagation, detection, and control can be fully integrated in one sample^14,15^. This on-chip platform has achieved effects such as THz antenna^16^, photonic crystals^17^, band-stop filer^18^, THz microcavities^19^, THz cloak^20^ and even designing research for THz metamaterials and negative refractive materials^21^.

However, it is challenging for simple LN waveguide to sever as a sensing or other utility applications, just like a PICs platform do, since the field intensity of the available THz source generated by LN slab is still low (the electric field intensity is generally several kV/cm)^22^. Although some researches attempted to solve the problem, for example, utilizing frequency-tunable multicycle THz pulses^23–25^, which can be excited by optical waveforms that are shaped temporally or spatially and has a narrow spectral range, will have excellent spectral brightness, most electric fields concentrate inside the crystal, not on the surface^14^. That brings a huge problem: how the inner THz energy interacts with the detected objects, which are set on the slab surface. In other words, how to make the inner THz energy come out of the LN crystal will be a necessary work.

In this paper we propose to use a pair of Au dipole antennas coupled to the subwavelength waveguide, which can efficiently convert propagating optical radiation to localized energy^26^, to provide large field enhancement and surface field localization. After depositing antennas on a LN subwavelength waveguide surface, we can achieve the antenna-induced frequency-dependent THz field enhancement between the gap of the antennas^16,27^. It has been demonstrated that dipole antennas enable several folds THz field enhancement in the gap between them^16^. However, simple dipole antennas, which have flat edges, cannot achieve the maximum field enhancement in the center of the gap, and they are also unbenefited for sensing, because the extremely small absorption cross sections of molecules require sufficiently focused power. Therefore, for optimal enhancement and sensing, we combined a bowtie structure with two strip antennas, and demonstrated the composite structure is conducive to gather more energy in the center of the gap.

**Figure 1:**
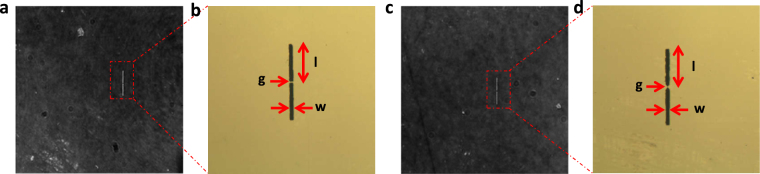
Images of the designed metal antennas. (**a**) Image of the SBAs (square bottom antennas) deposited on the LN crystal surface by phase contrast imaging. (**b**) An magnified view of (**a**) by polarizing microscope imaging. The single arm length is *l* = 112 μm, the width is *w* = 10 μm and the gap is *g* = 5 μm. (**c**) The image of the TBAs (tip bottom antennas) deposited on the LN crystal surface by the phase contrast imaging. (**d**) Magnified view of (**c**) by the polarizing microscope imaging. The single arm length is *l* = 112 μm (containing the tip part), the width is *w* = 10 μm and the gap is *g* = 5 μm.

Here, we designed two kinds of antennas on LN slab, both consist of two strips separated by a small gap and have the same arm length *l* (112 μm), width *w* (10 μm) and gap *g* (5 μm) with thickness of 100 nm, as represented in Fig. 1. Antennas with flat ends (Fig. 1(a) and (b)) serve as a reference, we call them the square bottom antennas (SBAs); while antennas consisting of two opposing tip-to-tip Au triangles (Fig. 1(c) and (d)) are called the tip bottom antennas (TBAs). We adopted a time-resolved phase contrast imaging system to visualize the propagation of THz waves and its interaction with the metal antennas, also to resolve electric fields both spatially and temporally^22,28,29^. Using our system we can show that TBAs enable more local *E*-field enhancement than SBAs, and quantitatively analyze the resonant frequencies of both antennas, which agree well with antenna theory and finite-difference time-domain (FDTD) simulations. Because time-resolved imaging relies on the electro-optic effect of LN crystal, evanescent fields that decay exponentially away from the crystal surface^14,21^, whose intensity are smaller than inner THz field^14^ but strongly influenced by the antennas, cannot be measured in our experiment. Here we took advantage of FDTD method to deal with it, also based on the FDTD simulations, we found that the energy of THz wave at the antenna gap is mainly localized on the surface of the LN. When the antenna is excited by a narrow-band THz source, whose center frequency is identical to antenna’s resonant frequency, it can achieve a superior effect. This opens the door for the sensing application, and also for THz spectroscopic analysis^30^ and interfacing the LN slab with THz devices, even research of electronic transitions in nanostructures^31,32^ like quantum wells or quantum dots^33^.

## Results

### Real-time imaging by time-resolved phase contrast

**Figure 2:**
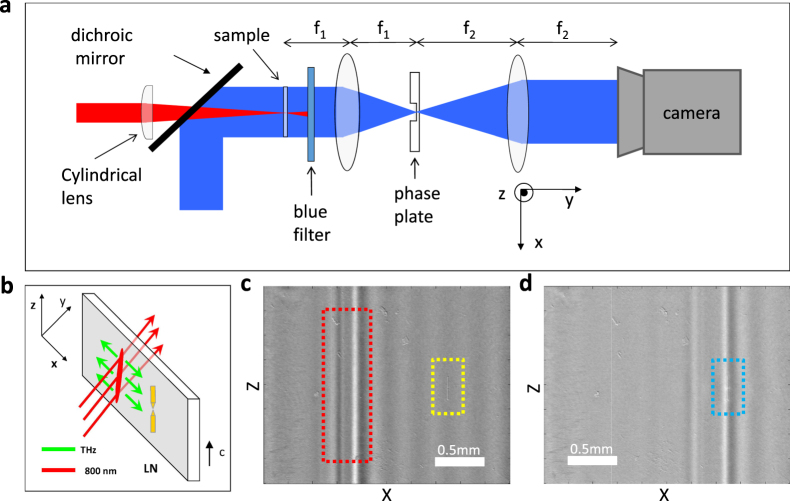
Experimental setup and images of THz waves obtained by phase contrast imaging method. (**a**) Schematic diagram of the experimental setup. The sample, a composite structure of THz antennas surfaced on a 50 μm thick LN slab, is imaged onto the CCD camera using two lenses. In the Fourier plane of the first lens, a phase plate is placed. The 800 nm pump beam (red) is linearly focused onto the sample and the 400 nm probe beam (blue) propagates perpendicular to the LN surface. The 800 nm pump and 400 nm probe are nearly collinear when they arrive at the sample. The focal lengths of the lens are *f*_1_  = 10 cm and *f*_2_  = 15 cm, respectively in our setup. (**b**) Pump geometry and coordinate system. The 800 nm pump beam (red) propagates orthogonally to the LN surface. The generated THz wave (green) propagates in the plane of the LN waveguide. (**c**) and (**d**) The different modes of THz waves propagate before and through the antennas. Red dotted lines show the THz waves, yellow dotted lines show the antennas, and blue dotted curves emphasize the visualization of enhancement in the gap of the antennas.

Figure 2(a) depicts our time-resolved phase contrast setup for the detection of THz wave propagating in the LN crystal and interacting with the antennas adhered to the LN surface. We use a cylindrical lens to produce a line source of THz waves which propagates almost perpendicular to the optical pump beam in the sub-wavelength waveguide, as shown in Fig. 2(b). Figure 2(c) and (d) show the propagation of different modes of THz waves (within the red dotted lines) and the process of their interaction with the antennas (within the yellow and blue dotted lines). A series of images can be obtained by changing the delay between the pump and probe pulses. We can comply these image sequences to form a movie (Media shown in electronic supplementary material) showing how the THz waves propagate.

### Resonant frequency and field enhancement

**Figure 3:**
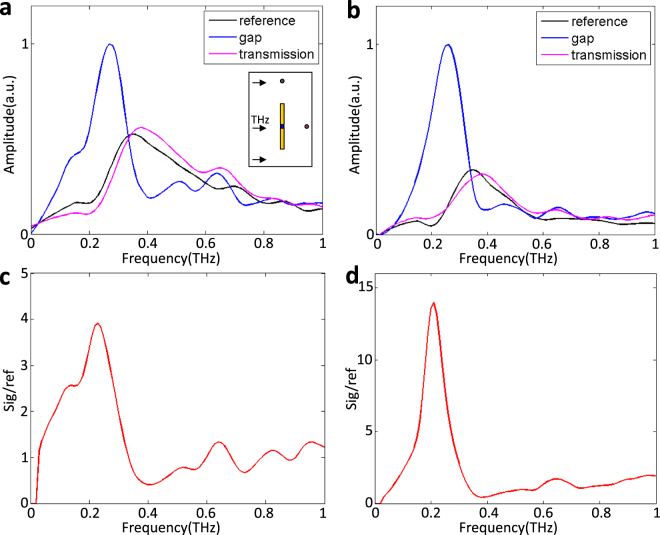
Frequency-domain characterization of the THz waves enhanced by the antennas. (**a**) and (**b**) are the spectral amplitude of each trace for the SBAs and the TBAs (blue gap spot, black reference spot and the magenta transmission spot in the inset of (**a**)), respectively. (**c**) and (**d**) exhibit the ratio of the gap signal to the reference corresponding to (**a**) and (**b**), respectively. It should be noticed that the amplitude at zero frequency should be zero in (**a**). This is caused by noise.

We spatially select three detecting points, shown in the inset of Fig. 3(a): a point in the antenna gap, a point far away from the antennas as reference signal, and a point soon after the THz wave propagating through the THz antennas. The amplitude spectrum of the three points, calculated by taking the Fourier transforms of the time domain signals (our time-resolved image sequence), are shown in Fig. 3. Figure 3(a) demonstrates the amplitude spectrum of the three points of the SBAs. According to the reference point (black curve), it can be seen that the excited THz wave has a broad-band spectrum with a central frequency of 0.36 THz. The peak in the region of the gap (blue curve) is at 0.26 THz. In addition, a small peak at 0.65 THz appears in the gap. The same method is used to analyze the TBAs, the amplitude spectrum of which is shown in Fig. 3(b). The reference point also has a central frequency of 0.36 THz, and the peak amplitude in the gap is at the same frequencies with SBAs, 0.26 THz and 0.65 THz. The ratio of the signal (in-gap) to reference, corresponding to the SBAs and TBAs, are shown in Fig. 3(c) and (d). The peak amplitude appears at same frequencies: 0.22 THz and 0.65 THz, which are considered as the resonant frequencies of the antennas. Moreover, the peak amplitude of the SBAs is enhanced 4-fold relative to the reference trace, while the peak amplitude enhancement of the TBAs is 14-fold.

### Surface localization proved by FDTD simulation

**Figure 4:**
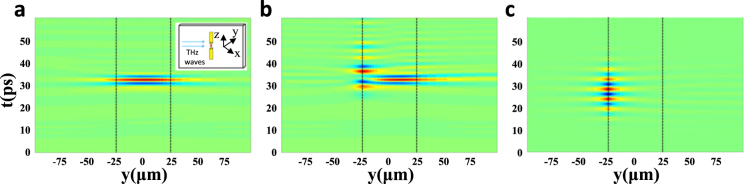
Simulation results by FDTD about surface localization. (**a**) The electric field intensity distribution when there is no antenna. (**b**) and (**c**) The electric field intensity distribution for antennas with tips. (**b**) Antennas excited by a broad-band THz source. (**c**) Antennas excited by a narrow-band source.

The simulations were performed for the TBAs structure set on the surface of the crystal, as shown in the inset of Fig. 4(a). The LN slab, whose thickness lies between *y* = ±25 μm, is marked by the black dotted lines in Fig. 4. We simulated the electric field intensity in *y* direction of the gap (*x* = *z* = 0, the center of the antennas) to find out how the antenna structure influences the distribution of THz wave. We drew the space-time plot of the propagating THz waves, and found that, as shown in Fig. 4(a), when there is no antenna, the electric field intensity distributes inside the crystal. While, as shown in Fig. 4(b), the THz source was set as a center frequency at the lowest resonant mode of the antenna and a 0.2 THz span. It is shown that the electric field intensity appears at *y* = −25 μm, where the antenna lies. And in Fig. 4(c), we set the THz source as a narrow-band radiation which has a 0.06 THz span, it shows that the most energy of THz wave is obviously localized on the surface of the LN crystal, which we expect to see. That means metallic antennas have the ability to realize strong field localization on the crystal surface, depending on the resonant frequency of the antennas and the property of the source.

## Discussion

**Figure 5:**
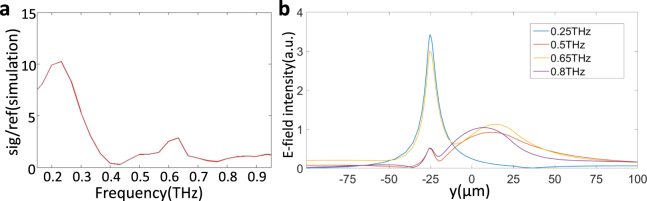
Simulation results by FDTD about resonant frequency. (**a**) The ratio of the gap signal and the reference signal for the TBAs. (**b**) THz energy with different frequencies as a function of *y*, when *x* = 0 and *z* = 0. −25 to 25 μm is the thickness of the crystal.

When the surface antennas driven by *z*-polarized THz waves, the charges move towards the ends of antennas. Each antenna can be described as a Fabry-Pérot resonator and thus a standing wave forms^34^. When the lengths of the antennas match with multiples of the half wavelength of THz wave, the antennas will be resonant and have maximum scattering efficiency at the gap. The resonant condition can be written as $l=\frac{m\lambda }{2}=\frac{mc}{2nf}$. Here *l* is the arm length of the antenna, *c* is the speed of light in vacuum, *n* is the refractive index of the surrounding medium, *f* is the resonance frequency and *m* is a positive integer that indicates the resonant mode. Under our experimental condition, the both ends of the antennas should have opposite charges, thus, only odd order resonances can be excited. The refractive index *n* represents the coupling between the LN subwavelength waveguide and the surface metal antennas, which is a rather complicated system to deal with. Therefore, we choose an approximate value *n* = 6.4 in the analysis as previously reported for similar structures^16^. In addition, though there are just a few differences between the SBAs and the TBAs, the arm lengths of both antennas are same. Hence, according to the resonance condition, the resonant frequencies can be calculated as 0.21 THz for the first mode (*m* = 1) and 0.63 THz for the second mode (*m* = 3). As can be seen from Fig. 3(c) and (d), the first two peak frequencies are 0.22 THz and 0.65 THz for both types of antennas. It indicates that the antenna with a tip make little difference on the resonant mode, while more charges are gathered around the cutting-edge of the TBAs, leading to a larger local field enhancement^35^. We also simulated the ratio of the gap signal to the reference signal of TBAs, shown in Fig. 5(a). The simulated broadband THz pulses are set in a range of 0.1–1 THz (a symmetrical shape, 0.55 THz for center frequency) and the polarization of electric field is parallel to *z* axis. Because the information of *y* direction extracted by the phase contrast method is an integral value, so here we sum the simulation values of all data points between *y* = ±25 μm. Comparing with the Fig. 3(d), we can infer that the resonant frequencies are well reproduced by this model, and the enhancement is approximately 10-fold, which is slightly smaller than the experimental result, maybe caused by the difference between the center frequencies of the THz source (0.36 THz is nearer the first resonant mode). It is concluded that the theoretical calculation and FDTD simulation results agree well with the resonant frequencies of peak enhancement in out experiment. What’s more, the needed resonant frequencies are able to be tuned by changing the length of the antennas.

In Fig. 4(a), we can see the evanescent fields that decay away from the interface and into the adjoining air. Meanwhile, as shown in Fig. 4(b) and (c), the antenna structure leads to the redistribution of the evanescent fields, also part of the inner THz energy coming out of the LN slab. Regrettably, they cannot be detected by time-resolved imaging method in our experiment^22^. In other words, the enhancement factors we calculated from our experiment are smaller than the real value, since they just represent the enhancement of the electric field inside the crystal. Fortunately, FDTD method can help us investigate these parts of energy. We set a point monitor, which lies in the gap of the antennas and slightly far away from the surface of the LN crystal, to compare the electric field intensity with that when there is no antenna. By a narrow-band source, the peak-to-peak electric field enhancement can reach 22-fold in our simulation. It is obviously larger than our experimental results. Therefore, it’s beneficial for many applications, especially sensing, because the measured molecules or medicines set on the crystal can interact with much surface-enhanced THz fields.

To further characterize the antennas’ effects, we set a frequency-domain monitor to record the results in FDTD model. Figure 5(b) shows the distribution of THz field intensity with different frequencies in *y* direction (also *x* = *z* = 0). We can conclude that when the frequencies of THz source near the resonant frequencies of the antennas, such as 0.25 THz (blue line) and 0.65 THz (yellow line), large amounts of energy is confined on the surface, and lower resonant mode brings better performance. Nevertheless, at non-resonant frequencies, such as 0.5 THz (brown line) and 0.8 THz (purple line), there is almost no contribution of antennas to surface field localization. That also explains why in Fig. 4(b), when the source is broad-band, some THz energy remains inside the crystal, but in Fig. 4(c) all energy comes out. Therefore, our TBAs can serve as a sensor to identify objects since they realize surface field localization, and for optimal effect, it is necessary to modulate the first resonant mode to the target frequency by changing the arm length of the antenna and the frequency of a narrow-band source. Fortunately, they’re all easy to be realized in the on-chip LN subwavelength waveguide.

In conclusion, we have designed and fabricated composite THz antennas, consisting of opposing tip-to-tip triangles and strips, which surfaced on a LiNbO_3_ subwavelength slab waveguide. By a time-resolved phase contrast imaging method, we experimentally demonstrated that the local field enhancement is achieved by the tips. FDTD methods were employed to reproduce the experimental data. Based on the experimental results and FDTD simulations, we show almost full energy of THz wave is localized on the surface of the LiNbO_3_ sample when its frequency is the same as antenna’s first resonant mode. Therefore, when coupling with the composite antennas, the LiNbO_3_ subwavelength slab waveguide becomes a more effective on-chip platform for THz applications, especially the research of material identification for THz-sensitive molecules or cells, as well as surface THz metamaterials and quantum dots.

## Methods

### Device fabrication

To fabricate these antennas, (**i**) a 2 μm thick layer of photoresist (RZJ304) was spin-coated onto a 50 μm thick LN substrate, (**ii**) the sample under the mask plate with the desired pattern was exposed to ultraviolet light via photolithography, (**iii**) the exposed photoresist was removed away with developer, (**iv**) a 100 nm layer of gold with a 10 nm adhesion layer of titanium were sputtered on the LN substrate by magnetron sputtering, (**v**) the intentional metal patterns were formed on the LN substrate by removing the unexposed photoresist with acetone. It should be noticed that the long axes of both antennas are parallel to the optical axis of the LN crystal.

### Experimental setup for THz wave generation and detection

The experiments were carried out with a Ti:sapphire regenerative amplifier (120 fs pulse duration, 800 nm central wavelength, 1 kHz repetition rate, 500 μJ/pulse). The laser pulses were separated into a pump beam (with 90% of initial energy) and a probe beam (with 10% of initial energy) to achieve a standard ultrafast pump-probe detection. The vertically polarized pump beam was routed through a mechanical delay stage and then focused into the sample (the LN slab with THz antennas) at normal incidence using a cylindrical lens (focal length 20 mm) to produce a line source of THz waves. For optimum signal, the polarization of the pump beam and the generated THz fields were all parallel to the *c*-axis of the LN crystal. The probe beam was frequency-doubled to 400 nm by a BBO crystal, spatially filtered to minimize spatial variation noise, and then expanded to be larger than the sample. The probe beam was nearly collinear with the pump beam by using a dichroic mirror. In order to exclude the affection of the second harmonic generation by the pump which has almost the same wavelength as the probe, we place a razor blade on the focal plane of the imaging lens to block this kind of signal. As shown in Fig. 2(a), the sample was imaged onto a CCD camera using two lenses. A phase plate was placed in the Fourier plane of the first lens. We fabricate the phase plate using a double sided fused silica optical flat (surface area 25 × 25 mm, thickness 1 mm). A 222 ± 5 nm layer of silicon dioxide (with refractive index of ~1.45 at 400 nm) was spin-coated onto the substrate. It was developed by removing a 35 × 35 μm area in the center of the plate using electron beam lithography. This led to a phase plate with an extremely flat surface that was transparent in the visible with a central square recessed by 222±5 nm, ~*λ*/4 for the 400 nm probe wavelength.

### Time-resolved phase contrast imaging

As LN is an electro-optic crystal, the THz electric field generated through ISRS can modulate the refractive index of the host crystal when propagating through the sample. The time delayed probe pulses that illuminated the whole sample experienced a spatial phase modulation proportional to the change of refractive index. Then the phase plate can convert the modulated phase pattern to amplitude information which can be recorded by the CCD camera. The relationship between the induced THz electric field and the phase shift can be described as: ${\rm{\Delta }}\varphi (x,z,t)=2\pi \frac{L}{\lambda }{\rm{\Delta }}n(x,z,t)=2\pi \frac{L}{\lambda }\frac{{n}_{eo}^{3}{r}_{33}}{2}{\rm{\Delta }}{E}_{THz}(x,z,t)$. Here *L* is the slab thickness, *λ* is the probe wavelength, *r*_33_ is the appropriate electro-optic coefficient, *n*_*eo*_ is the extraordinary index of refraction of LN for the probe, and *E*_*THz*_ is the average THz field experienced by the probe pulse as it propagates through the LN in the *y* direction. Therefore, it is able to quantitatively calculate the THz fields.

### FDTD methods

FDTD simulations were performed for the system used in the experiment, using a commercial package to solve Maxwell’s Equations (Lumerical). The LN slab, whose thickness lies between *y* = ±25 μm, is modeled with an ordinary refractive index *n*_*o*_ = 6.4 and an extraordinary one *n*_*e*_ = 5.11. The simulations are performed for the TBAs structure set on the surface of the crystal. In particular we study a pair of antennas with volume of a single arm of 10 × 0.1 × 112 (107 μm for strip and 5 μm for tip) μm^3^ and gap size of 5 μm. The center of the antennas is situated at (0, −25, 0). A non-uniform three-dimensional discretization mesh of 0.5 × 0.1 × 0.5 μm^3^ is used in the vicinity of the gap. We choose the Perfect Electrical Conductivity (PEC) for gold because Au being very close to a perfect metal in THz frequency range.

## Electronic supplementary material

Supplementary information
